# Fish-Derived Antifreeze Proteins and Antifreeze Glycoprotein Exhibit a Different Ice-Binding Property with Increasing Concentration

**DOI:** 10.3390/biom10030423

**Published:** 2020-03-09

**Authors:** Sakae Tsuda, Akari Yamauchi, N. M.-Mofiz Uddin Khan, Tatsuya Arai, Sheikh Mahatabuddin, Ai Miura, Hidemasa Kondo

**Affiliations:** 1 Graduate School of Life Science, Hokkaido University, Sapporo 060-0810, Japan; a19059v-akw-v7@eis.hokudai.ac.jp (A.Y.); mofizchemdu@gmail.com (N.M.-M.U.K.); tatarai0926@gmail.com (T.A.); h.kondo@aist.go.jp (H.K.); 2 Bioproduction Research Institute, National Institute of Advanced Industrial Science and Technology (AIST), Sapporo 062-8517, Japan; a.miura@aist.go.jp; 3 OPERANDO Open Innovation Laboratory, National Institute of Advanced Industrial Science and Technology (AIST), Tsukuba 305-8563, Japan; 4 Department of Nutrition and Food Engineering, Daffodil International University, Dhanmondi, Dhaka 1207, Bangladesh; mahatab.chem@gmail.com

**Keywords:** antifreeze protein, hydration, ice-binding, thermal hysteresis, solubility, structure

## Abstract

The concentration of a protein is highly related to its biochemical properties, and is a key determinant for its biotechnological applications. Antifreeze proteins (AFPs) and antifreeze glycoproteins (AFGPs) are structurally diverse macromolecules that are capable of binding to embryonic ice crystals below 0 °C, making them useful as protectants of ice-block formation. In this study, we examined the maximal solubility of native AFP I–III and AFGP with distilled water, and evaluated concentration dependence of their ice-binding property. Approximately 400 mg/mL (AFP I), 200 mg/mL (AFP II), 100 mg/mL (AFP III), and >1800 mg/mL (AFGP) of the maximal solubility were estimated, and among them AFGP’s solubility is much higher compared with that of ordinary proteins, such as serum albumin (~500 mg/mL). The samples also exhibited unexpectedly high thermal hysteresis values (2–3 °C) at 50–200 mg/mL. Furthermore, the analysis of fluorescence-based ice plane affinity showed that AFP II binds to multiple ice planes in a concentration-dependent manner, for which an oligomerization mechanism was hypothesized. The difference of concentration dependence between AFPs and AFGPs may provide a new clue to help us understand the ice-binding function of these proteins.

## 1. Introduction

Concentration dependence is a significant factor for all protein studies and their applications. Protein concentration varies from almost completely insoluble to hundreds of milligrams per milliliter [1,2]. An example of an insoluble protein is crambin, whereas serum albumin is one of the highly dissolved proteins (>500 mg/mL) [3]. Both intrinsic and extrinsic factors are known to affect solubility. Examples of intrinsic factors are the primary to tertiary structures of proteins with a hydration shell, although their relationship with solubility is not perfectly understood. The extrinsic factors include ionic strength, pH, temperature, and buffer detergents. In this study, we evaluated the solubility limit of native antifreeze proteins (AFPs) and antifreeze glycoprotein (AFGP) using 4 °C water, and examined how their ice-binding activity responds to the change in concentration. Distilled water was used as the solvent in all experiments. The X-ray structural coordinates including the hydration waters are available for AFPs [4,5].

AFPs and AFGP have been purified from various cold-adapted fishes living in northern midlatitude waters [6]. These include AFP I from barfin plaice (*Liposetta pinnifasciata*) [7], AFP II from longsnout poacher (*Brachyopsis rostratus*) [8], AFP III from notched-fin eelpout (*Zoarces elongatus Kner*) [9], and AFGP from saffron cod (*Eleginus gracilis*) [10]. A technique to obtain massive amounts of these proteins has been developed, which utilizes the muscle homogenate of each fish as a source material [11]. For AFP I–III, the obtained samples were found to consist of a mixture of the isoforms, in which the primary sequence of the dominant species was determined. Three-dimensional (3D) structures were also determined for each AFP isoform. For example, AFP I is an alanine-rich amphipathic α-helical polypeptide (Mw = 3.5 kDa), AFP II is a disulfide-bond-rich globular protein exhibiting high structural similarity with a carbohydrate-recognition domain of C-type lectin (Mw = 14 kDa), and AFP III is another globular protein composed of twisted loops folded into triple-strand β-sheets (Mw = 6.5 kDa) [10]. The AFGP consists of tripeptide repeats (Ala–Ala–Thr)_n_ (*n* = 4–50), in which C_β_ of Thr is glycosylated with a disaccharide β-d-galactosyl-(1→3)-α-*N*-acetyl-d-galactosamine [12]. Hence the molecular weight of AFGP ranges from 2.6 kDa (*n* = 4) to 33.7 kDa (*n* = 50). The AFGP purified from saffron cod consists of at least 18 distinct isoforms, in which a few amino acids are replaced with Arg or Pro [13]. Although X-ray structure was not determined for AFGP, a structural motif called “polyproline type II helix” was hypothesized on the basis of its circular dichroism (CD) spectra and solution nuclear magnetic resonance (NMR) data [14].

When approximately 10 mg/mL solution of AFP or AFGP is placed in a freezer for example, neither of them turns into a normal ice block, but instead transforms into an assembly of numerous dispersive single ice crystals [10,11]. The growth of each crystal is inhibited, as the antifreezes adsorb onto each crystal surface via an “adsorption–inhibition mechanism” [15]. The efficiency of ice growth inhibition is evaluated using thermal hysteresis (TH), the difference between the melting and freezing points of a solution (*T*_m_ and *T*_f_, respectively), which is controlled according to the types and concentrations of AF(G)Ps [4,5,6]. In previous studies, 0.6–1.0 °C of TH was determined for AFP I–III at the concentration of approximately 10 mg/mL, although their solubility limit was not closely examined. The TH values of only 0.01–0.03 °C were evaluated for AFP mimetics, such as 50 mg/mL solution of polyvinyl alcohol (PVA) [16]. A relatively high TH value of 1.4 °C was evaluated for an approximately 7.5 mM (25 mg/mL) solution of AFP I [17]. A lower solubility was suggested for AFP III, so that a hydrophilic tetrapeptide sequence KDEL was appended to the C terminus of the AFP III isoform for NMR studies [18]. This mutant’s TH value is almost the same as that of wild-type (~0.8 °C at 6 mg/mL). In contrast, approximately 1.2 °C of maximal TH value was evaluated for a 40 mg/mL solution of AFGP, suggesting its superior solubility [12].

A recent study showed that a native AFP I sample purified from barfin plaice is extremely soluble (~650 mg/mL), and exhibited high TH activity (3.0 °C) at 200 mg/mL [7]. The study showed that this AFP I binds to only a limited area of an ice crystal at lower concentrations (<0.01 mg/mL); however, it expands the target area to the whole crystal surface when the concentration is increased up to 0.1 mg/mL. This prompted us to question whether high water solubility is a common property of fish AFPs and AFGP, and how their ice-binding activity changes in response to the increase in concentration. Computational approaches and structural biology pointed out that an ice-binding site (IBS) of AFP accompanies a unique hydration shell including some waters organized into an ice-like arrangement [19,20,21]. Such IBS formation was suggested for AFPs from bacteria [22], fungi [23], and fishes [24], but not those from insects [25]. The ice-like waters were hypothesized to merge with, and freeze to, an intrinsically disordered water layer constructing the surface of ice, leading to AFP–ice complex formation [26]. Davies and colleagues [22] named this mode of AFP function the anchored clathrate water mechanism. The present study examined the water solubility, TH value, and fluorescence-based ice plane affinity (FIPA) of each sample, and results are considered in relation to the anchored clathrate water mechanism.

## 2. Materials and Methods

### 2.1. Preparation of Antifreeze Proteins and Antifreeze Glycoprotein

The fish species barfin plaice (*Liposetta pinnifasciata*), longsnout poacher (*Brachyopsis rostratus*), notched-fin eelpout (*Zoarces elongatus* Kner), and saffron cod (*Eleginus gracilis*) were collected by Nichirei Corporation. Size exclusion chromatography and anion exchange chromatography were successively performed for the prepared crude sample powders with a Sephadex G-25 size-exclusion column (XK 50/30, 500 mL; GE Healthcare Life Sciences, Pittsburgh, PA, USA) and DEAE Sepharose anion-exchange column (XK 50/20, 80 mL; GE Healthcare), respectively [7]. The purified samples were dialyzed against Milli-Q water for three overnights, and then lyophilized for frozen storage. The purity of the final product was checked with 15% SDS–PAGE using a minislab electrophoresis kit (AE-6500; ATTO Corp., Tokyo, Japan).

### 2.2. Evaluation of Protein Concentration

**Figure 1:**
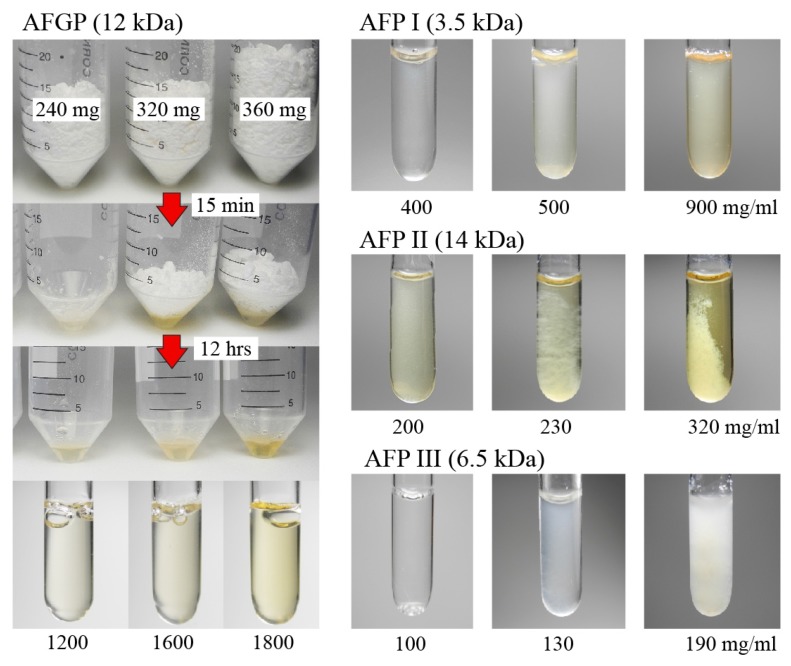
Solubility limit evaluation for native antifreeze proteins (AFP I–III) and antifreeze glycoprotein (AFGP) samples. For AFGP, 240, 320, and 360 mg each of the lyophilized powder was dissolved with 200 µL distilled water precooled to 4 °C. After a 12 h incubation at 4 °C, each solution was transferred to an NMR tube (Ø = 5 mm) so images can be captured, none of which showed any precipitant. The evaluation was similarly performed for the others by dissolving 40, 60, 80, 100, 180 mg of AFP I; 16, 22, 40, 46, 64 mg of AFP II; and 10, 15, 20, 26, 38 mg of AFP III with 200 µL of 4 °C chilled water. A critical concentration (mg/mL) that generates no significant precipitant was determined as the solubility limit.

The lyophilized powder of each AFP I–III and AFGP was weighed and dissolved with 200 µL of distilled water in a 50 mL centrifuge tube. The respective samples were then placed into NMR tubes (Ø = 5 mm), where they were left overnight at 4 °C, and photographs of the samples were taken (Figure 1). Approximately 400 mg/mL (AFP I), 200 mg/mL (AFP II), 100 mg/mL (AFP III), and >1800 mg/mL (AFGP) of the concentration limit were evaluated at 4 °C; results showed that no significant precipitants were produced below these concentrations. The weight-base evaluation was further verified using a fluorescence method (Qubit Protein Assay Kit; Thermo Fisher Scientific, Waltham, MA, USA). We prepared 2–15 µg/mL protein samples to be reacted with a dye reagent provided in this kit. The correlation between protein concentration and the fluorescence emission was calibrated by standard protein solution supplied by the kit. We confirmed that the estimated protein concentration by the kit was consistent with weight-based concentration by applying another protein standard (chicken lysozyme).

### 2.3. Analysis of Fluorescence-Based Ice Plane Affinity

For the FIPA experiment, several single ice crystals with a cylindrical shape (Ø = 2–3 cm) were initially prepared within the plastic pipes (l = 4 cm) according to a previously described procedure [27,28,29]. After determination of their *c*-axis on a polarizer, each cylindrical ice was half-cut and mounted on a hollow copper tube (Ø = 15 mm), in which −0.8 °C coolant was circulated by a refrigerant pump (Hitachi AMS-007; Hitachi, Tokyo, Japan). The cylindrical ice crystal was then immersed in distilled water for overgrowth to be changed into a hemispherical shape. We prepared many such spherical single ice crystals, and immersed each crystal in a solution of fluorescent AFP II or AFGP, whose concentration was adjusted between 0.01 and 0.1 mg/mL. After 2 h of incubation of each crystal in a solution, the fluorescence emission or the FIPA pattern was observed under UV light. After capturing the snapshots, a six-sided star mark was created in the center of each spherical ice with the ice-pitting protocol [27], which determined the *a*_1–3_-axes of each single ice crystal.

### 2.4. Ethical Approval

All methods were carried out in accordance with relevant guidelines and regulations of National Institute of Advanced Industrial Science and Technology (AIST), Japan. All experiments involving animals were conducted with approved methods designated in Guidelines for Proper Conduct of Animal Experiments, Science Council of Japan (Low No. 105, 1973).

## 3. Results and Discussion

### 3.1. Solubility Limit Evaluation for Native Antifreeze Proteins and Antifreeze Glycoprotein

Sample powders of AFP I–III and AFGP of 20–30% purity were obtained from Nichirei Corporation (Tokyo, Japan), and were extracted from the muscle homogenates of barfin plaice, longsnout poacher, notched-fin eelpout, and saffron cod, respectively [11]. Size exclusion chromatography followed by anion exchange chromatography were performed on 6 g of each crude powder according to previously described procedures [7,8,9,10,11], which yielded approximately 1500, 200, 130, and 1600 mg of lyophilized powders of AFP I–III and AFGP, respectively. Then, chromatography was performed again to obtain the gram order of the samples to be analyzed with high-performance liquid chromatography, which gave us several isoform fractions. The bipyramidal ice crystals were observed for each sample as a typical sign of their ice-binding ability [30]. Their electrophoretograms with 15% tricine sodium dodecyl sulfate–polyacrylamide gel electrophoresis (SDS–PAGE) exhibited a typical major band (supporting information S1) attributable to their isoform mixture [9,11,31]. The analysis of SDS–PAGE bands using Image-J (https://imagej.nih.gov/ij/) [32] enabled us to know that our samples are purified to 93–97% homogeneity.

The consistency between the weight-base and the fluorometer-base concentrations was verified for AFP I–III within an error range of 1–12% (Supporting Information S2). An ultimately concentrated solution of AFGP (>1800 mg/mL) generated no precipitant but exhibited significant viscosity similar to that of high molecular weight polymers; they may become viscous by means of a strong internal friction between the randomly coiled swollen polymers with surrounding solvents [33]. For proteins, precipitants and/or viscosity of the condensed solution have been assumed to originate from more complex factors, such as hydration, surface charge, hydrophobicity, and 3D structure, as they alter the manner of protein–protein interaction [1,2]. It is significant that when the AFP I solution was incubated at 25 °C for 12 h, it became highly viscous and changed into a gel state. This AFP I gel was changed into a solution state when the temperature was decreased to 4 °C. The viscosity of the AFGP solution became more significant, and the solubility of AFP II and III was lowered to 20–30 mg/mL when they were incubated at room temperature. These results suggest that native AFP I–III and AFGP samples are highly soluble only when they are dissolved in chilled water. An AFP molecule generally forms a hydrophobic surface that locates several polar residues to construct the ice-binding site. The temperature increase theoretically strengthens the hydrophobic interaction [1,2,3] between AFPs in solution, which might be a reason of the precipitation and lower solubility of AFPs and AFGP at higher temperature.

### 3.2. Thermal Hysteresis Measurement for the Native Samples

**Figure 2:**
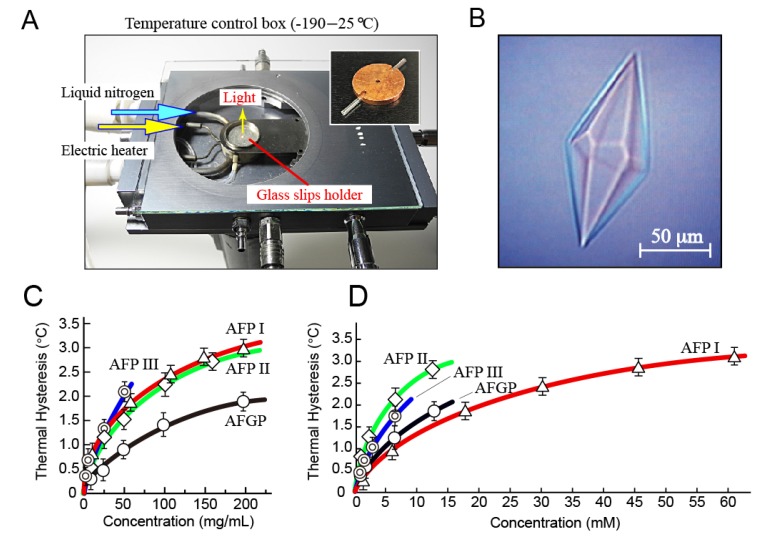
Thermal hysteresis evaluation for native AFP I–III and AFGP samples up to 200 mg/mL. (**A**) A temperature control box to be placed on a photomicroscope stage, which allowed observation of freezing and melting processes of a solution. Approximately 1 µL of the solution is loaded into a capillary tube soaked in a copper disk (shown in a small picture) whose temperature is manipulated on a glass slips holder with ±0.2 °C accuracy using liquid nitrogen and an electric heater. (**B**) An example of single ice crystal whose morphology was changed into a hexagonal bipyramid by AFP adsorptions. The image was observed for 0.2 mg/mL solution of AFP III. (**C**) Weight-base concentration dependence of thermal hysteresis (°C) of AFP I–III and AFGP. It was obtained for up to 50 mg/mL for AFPIII and 200 mg/mL for the others, as one single crystal was difficult to prepare above these concentrations. (**D**) Molar-base concentration dependence of thermal hysteresis (°C) of AFP I–III and AFGP. For these plots, 3300; 14,000; 6500; and 12,000 Da were assumed as the average molecular weights for the native AFP I–III and AFGP samples, respectively.

TH is defined as the difference between the *T*_m_ and *T*_f_ of an AFP solution [15,34]. The |*T*_f_| value is also called freezing-point depression. Differential scanning calorimetry (DSC) [35] and sonocrystallization method [36] were the methods used to determine |*T*_f_| via detection of the latent heat emission that originates from the number of crystals generated at the moment of freezing. In contrast, a method that uses a photomicroscope system equipped with a cooling stage to determine the growth-initiation temperature and melting temperature of a single ice crystal was also introduced [37,38]. The two respective temperatures are equal to the *T*_f_ and *T*_m_ of an AFP solution [38]. The TH value (i.e., TH = *T*_m_ − *T*_f_) is the temperature range in which an AFP-adsorbed single ice crystal neither grows nor melts. The TH was therefore used as a parameter to evaluate the AFP’s ice-binding ability. It should be noted that the cooling rate (°C/min) and ice crystal size (µm) need to be adjusted before one can compare the TH value between samples. Such a photomicroscopic method was used in this study, for which a key device is shown in Figure 2A. This is a temperature control box (type 10002L; Linkam Scientific, London, UK) covered with a glass plate to be placed on a photomicroscope stage. The temperature of the glass slips holder (Figure 2A) was controlled to be between −198 and 600 °C with an accuracy of ±0.2 °C by mixing the use of liquid nitrogen and an electric heater. A 0.8 µL AFP sample was soaked into a capillary tube (Ø = 0.92 mm) to be set into a homemade copper holder (shown in Figure 2A). This was then set into the portion of the glass slips holder to observe the process of sample freezing and/or melting under the beam of the light, thus allowing TH evaluation.

When the temperature of a sample was constantly lowered on this device (−20 °C/min) (Figure 2A), the photomicroscope view was suddenly darkened at approximately −15 °C, implying that the solution was flash frozen to form a polycrystalline state composed of numerous single ice crystals [39]. They were tightly assembled together at −15 °C, while gradually melting to form their dispersion state with the increase in temperature. We stopped the temperature increase before all ice crystals melted at slightly below 0 °C, and then carefully repeated the temperature increase and decrease so as to isolate a single ice crystal of approximately 20 µm in diameter. We then restarted the constant lowering of temperature with a very slow cooling rate (−0.1 °C/min), which soon changed the single ice crystal morphology into a hexagonal bipyramid (Figure 2B). This ice bipyramid neither grew nor melted within the temperature gap between *T*_m_ and *T*_f_, or the TH value, as AFPs bind on to this bipyramid to terminate its further growth. If the lowering of temperature exceeded the *T*_f_ point, a bursting growth occurred from a portion that is not strongly protected by AFPs. Although such portions were generally two tips of the ice bipyramid for AFP I–III and AFGP, they were different for the other types of AFPs from insects, fungi, and bacteria. For a reference solution (water), only a slight temperature change affected the growing and melting of a rounded disk-shaped ice crystal, which indicates the equality of Tm and Tf, implying no TH activity [38].

The TH value was determined for the AFP I–III and AFGP samples (Figure 2C,D). The measurement was performed at least three times, and their averaged values are plotted with error bars. A profile of each concentration dependence showed a known hyperbolic-type curve [40,41]. Data were obtained only up to 200 mg/mL for AFP I–II and AFGP, and up to 50 mg/mL for AFP III, because preparation of a single ice crystal in these solutions became extremely difficult above these thresholds. As shown, the maximal TH of 3.0, 2.9, 2.3, and 1.9 °C were evaluated for AFP I–III and AFGP, respectively, which are considerably higher than the known maximal values (e.g., 0.5–1.5 °C) [41]. Note that 100–1000 mg of the purified samples were necessary for these experiments (Figure 1). To obtain such large amounts of AFP and AFGP, a method was developed that includes AFPs into a growing ice crystal with ice-affinity protocol [42,43], as well as a method that utilizes fish muscle homogenates [7,8,9,10,11]. The latter method was used to obtain the present samples.

Kinetic analyses suggest that AFPs are associated with the ice surface within the hysteresis gap [15,44]. Kristiansen and Zachariassen [45] assumed that TH activity is likely to be determined by the density of AFPs on the ice surface. A reduction of the solubility of AFPs increases the density of AFPs within the ice–water interface, which was thought to enhance TH activity. Many small aggregates of AFP I were observed at the interface below 0 °C [46]. The inverse order of water solubility is as follows: AFP III ≥ AFP II > AFP I > AFGP (Figure 1), which is almost proportional to the order of the weight-base TH, AFP III > AFP I ≥ AFP II > AFGP (Figure 2C), in accordance with the previous assumption. The solubility order, however, is not consistent with the molar-base TH order, AFP II > AFP III > AFGP > AFP I (Figure 2D). The discrepancy may suggest that TH value is affected by an additional mechanism, such as ice-binding specificity of each AFP species.

### 3.3. Analysis of Fluorescence-Based Ice Plane Affinity (FIPA) for the Native Samples

A technique has been developed to determine the ice-binding specificity of AFPs through the observation of their FIPA on a single ice crystal hemisphere (Ø = 3 cm) [27,28]. The list below shows AFPs and their target ice planes:

(a) Ca^2+^-dependent AFP II, AFP III, β-helical AFPs—First prism plane;
(b) Sculpin AFPI, Ca^2+^-independent AFP II, β-helical AFPs—Second prism plane;
(c) Flounder AFP I, AFP III, β-helical AFPs—Pyramidal plane;
(d) Flounder AFP I dimer (Maxi), β-helical AFPs—Basal plane.

**Figure 3:**
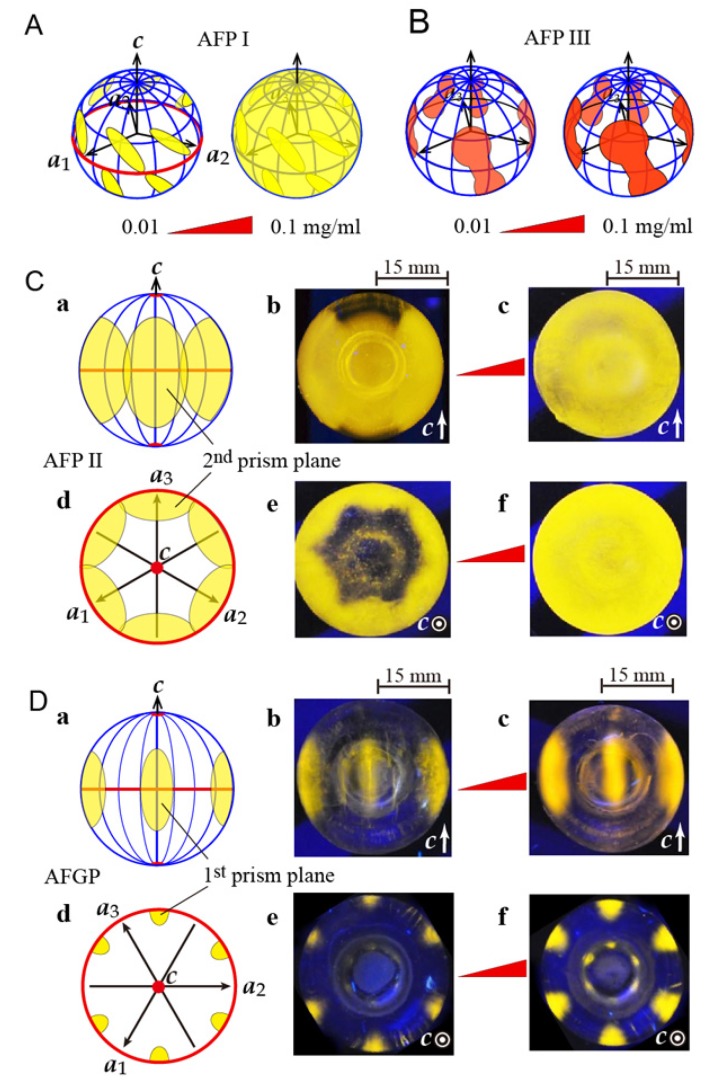
Fluorescence-based ice plane affinity (FIPA) of the native AFP I–III and AFGP samples. (**A**) Illustration of the known FIPA pattern of AFP I. Inclined ellipses implying the AFP I binding to ice pyramidal planes were overcast by an entire illumination that progressed on the spherical ice after increasing the concentration from 0.01 to 0.1 mg/mL. (**B**) Illustration of the known FIPA pattern of AFP III. Dumbbell patterns implying the AFP III binding to both first prism and pyramidal planes were not overcast. (**C**) FIPA pattern of AFP II observed at the concentration of 0.01 mg/mL (b,e) and 0.1 mg/mL (c,f). Panels a–c are the views parallel to the second prism planes and panels d–f are those normal to these planes. Images b and e are illustrated as a and d, respectively, to indicate the location of the ice planes and unit axes. The patterns showing the second prism plane binding (b,e) are overcast by entire illumination up to 0.1 mg/mL (e,f). (**D**) FIPA pattern of AFGP observed at the concentration of 0.01 mg/mL (b,e) and 0.1 mg/mL (c,f). Panels a–c are the views parallel to the first prism planes and panels d–f are those normal to these planes. The patterns showing the first prism plane binding (b,e) are not overcast up to 0.1 mg/mL (e,f).

Each target plane was determined according to the illumination area called “FIPA pattern” on the spherical ice crystal, to which fluorescent AFPs adsorbs. A wider FIPA pattern implies an extensive AFP adsorption, which leads to an effective ice growth inhibition and a high TH activity [24]. Figure 3A,B illustrate the known FIPA patterns observed for the fluorescent AFP I and III, respectively [7]. The inclined ellipses were observed for AFPI at upper and lower sides of the equator (Figure 3A, left) when its concentration was 0.01 mg/mL. This pattern was attributed to AFP I binding to ice pyramidal planes. Meanwhile, the ellipses were overcast by illumination, which progressed entirely on the spherical ice surface with increasing AFP I concentration (Figure 3A, right). Such change was completed up to 0.1 mg/mL, showing that AFP I is capable of binding to multiple ice planes. AFP I was hence thought to possess an ability to expand its target area from the local to the entire surface of an ice crystal. No such concentration dependence was observed for AFP III (Figure 3A, right) [7], which is bound to a dumbbell-like area composed of first prism and pyramidal planes [24].

The present study examined the concentration dependence of the FIPA pattern for AFP II (Figure 3C) and AFGP (Figure 3D). In Figure 3C, the upper row (a–c) shows the side views of the FIPA pattern of AFP II, whereas the lower row (d–f) shows the views looking down from the polar area. Images b and e are the snapshots of the spherical ice soaked in the 0.01 mg/mL AFP II solution, whereas images c and f show the moment when the ice crystal was soaked in the 0.1 mg/mL solution. The unit axes (*c*- and *a*_1_- to *a*_3_-axes) of the spherical ice crystal are indicated in Figure 3C-a and -b, where the area on the *a*_1_- to *a*_3_-axes are the second prism planes, and the polar regions correspond to the basal planes [28]. As shown, illumination was observed on *a*_1_–*a*_3_ axes symmetrically but not in the polar regions. The data hence indicate that AFP II binds to the second prism plane but not to the basal plane at the concentration of 0.01 mg/mL. The spherical ice is illuminated entirely with the increase in AFP II concentration up to 0.1 mg/mL. This indicates that AFP II expands its target area to the entire ice surface including the basal plane, similar to AFP I [7]. Note that the observed difference of the brightness between the images (Figure 3) should be evaluated only qualitatively.

Figure 3D shows the FIPA pattern examined for AFGP from saffron cod. The ordinary AFGP sample is a mixture of repetitive peptides represented by (Ala–Ala–Thr)n (*n* = 4–50) whose Thr sidechain is modified with a disaccharide [12]. The ordinary AFGP reacts poorly with a fluorescent detergent, as Ala and Thr have no NH^3+^ group, whereas saffron cod AFGP contains Arg [13]. This guanidino group reacts with fluorescence 5(6)-TAMRA-X SE (Thermo Fisher Scientific), which enabled us to perform the FIPA experiment. The upper row (a–c) of Figure 3D shows the side views of the illumination observed for our AFGP, and the lower row (d–f) shows the views looking down from the basal plane. Images b and e were captured for the 0.01 mg/mL solution of AFGP, whereas images c and f were those for the 0.1 mg/mL solution. As shown, the area sandwiched between the two *a*-axes were illuminated on the ice sphere Figure 3D(b,e), which were assigned to the first prism planes. The size of the AFGP-bound area was rather small compared with the pattern observed for AFP II (Figure 3C). The illumination in Figure 3D(c,f) observed for the 0.1 mg/mL AFGP solution exhibited more brightness compared with 0.01 mg/mL, but they are not overcast by entire illumination. The data therefore show that AFGP is unable to expand its target area to whole ice planes, in contrast to AFP I and II (Figure 3A,C). Knight et al. [47] originally developed a technique called “ice etching” to visualize the AFGP’s target plane on an ice crystal hemisphere without using fluorescence, which also suggested the AFGP binding to the first prism plane. They assumed that AFGP is folded into a polyproline II helix with a periodicity of 9.31 Å, which is almost two-fold of the oxygen atom spacings (4.51 Å) constructing the first prism plane. This lattice matching between the charged atoms of AFGP and frozen waters was assumed for its binding ability to this plane.

**Figure 4:**
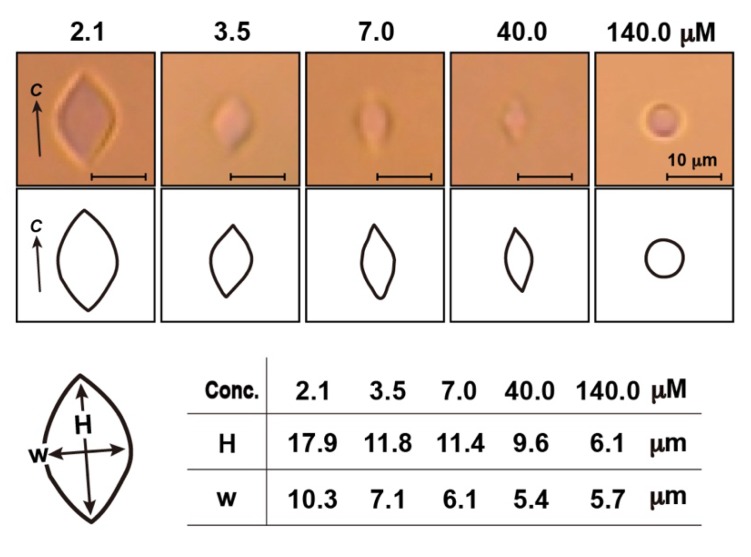
Change in the photomicroscopic image of a single ice crystal with the increase in concentration of AFP II, in which scale bars represent 10 µm. Illustrated interpretations are indicated for each small image whose edge is hardly detected. The crystallographic *c*-axis of the lemon-shaped crystal is indicated in the left panel. For concentrations 2.1, 3.5, 7.0, 40.0, and 140.0 µM of AFP II, the corresponding aspect ratio (W/H) is 0.58, 0.60, 0.54, 0.56, and 0.94, respectively.

Figure 4 shows the photomicroscopic images of an ice crystal observed for the 2.1–140.0 µM solutions of AFP II. In contrast to the bipyramidal ice crystal observed for ordinary fish AFPs (e.g., Figure 2B), AFP II modified the crystal into a rounded lemon-like morphology, as can be best identified for a large crystal created in the highest concentration of AFP II (Figure 4, left). This lemon-shaped crystal further supports the binding of AFP II to multiple ice planes, because such a crystal was generally observed for hyperactive AFPs that were capable of binding to whole ice planes [37]. As shown in Figure 4, both the height and width of the lemon-shaped crystal became shorter with the increased AFP II concentration. These observations are highly similar to the results obtained for AFP I from barfin plaice, for which a small percentage of the proteins was shown to form dimers and tetramers in a concentration-dependent manner [7].

### 3.4. Oligomerization Hypothesis for Type II Antifreeze Protein

The present experiments showed that not only AFP I [7], but also AFP II, expands the target area to multiple ice planes with the increase in concentration (Figure 3C). For AFP I, oligomerization was hypothesized to assemble plural IBSs to acquire additional ability to bind multiple ice planes and to minimize the crystal size (Figure 4). Although the oligomerized variant of AFP II has not been identified so far, it has been shown that the C-type lectins, the protein family exhibiting a high structural homology to AFP II, inevitably undergo oligomerization to exert the cell–cell adhesion function or the immune response to pathogens and apoptosis [48,49,50]. Walker et al. [49] determined the X-ray crystal structure of a 135-residue C-type lectin from rattlesnake venom (PDB code, 1JZN) to show that five lectins are assembled side-by-side to form a disk-shaped pentamer, and two of them are stacked with a slight distortion to complete the lectin decamer. A total of 10 carbohydrate recognition sites are located on the rim of the decamer, for which multivalent interactions and the ability to promote receptor cross-linking and cell-aggregation were assumed. By utilizing this decamer as a structural template, we modeled an oligomerized form of AFP II and considered its relevance to the ice-binding property.

**Figure 5:**
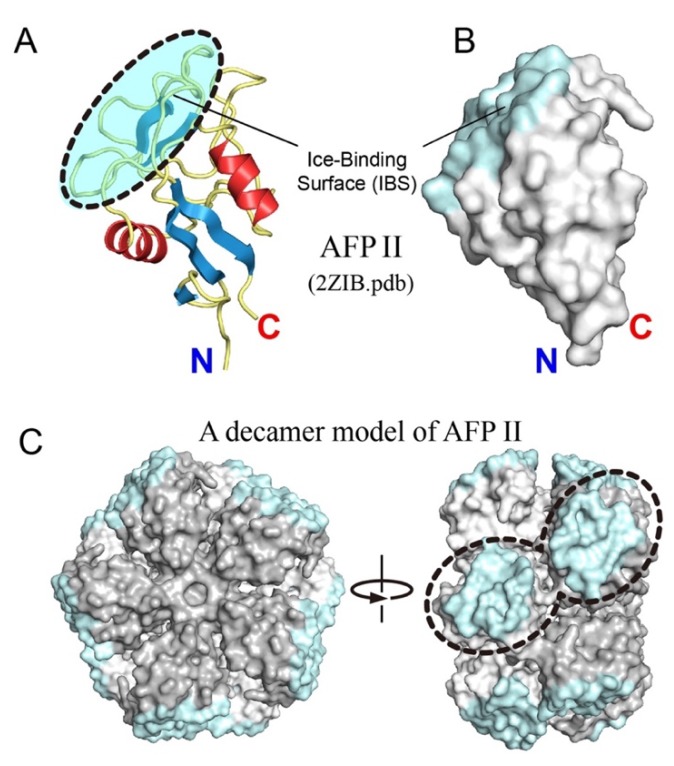
Prediction of a decamer model of AFP II. (**A**) A ribbon representation of the crystal structure of AFP II (2ZIB.pdb) whose α-helices, β sheets, and loops are shown in red, blue, and yellow, respectively. A cyan region shows the putative ice-binding site (IBS) contributed by I58, C86, K89, N91, I93, S95, M99, Q100, T102–D106, C108, D110–H118. (**B**) Surface representation of AFP II whose orientation is the same as (**A**). (**C**) A structural model of AFP II created by using a decamer structure of rattlesnake venom lectin (RSL) (1JZN.pdb) as a template. A front view of the model (left) shows that the IBSs are located at the rim of the decamer disk. The side view (right) shows that two imperfectly aligned IBSs are proximal to each other.

The present AFP II is a 127-residue Ca^2+^-independent species obtained from longsnout poacher (*B. rostratus*) (2ZIB.pdb) [8], in which 115 residues share the sequence identity with the rattlesnake venom lectin (RSL) (1JZN.pdb) [49]. The AFP II consists of two α-helices and eight β-strands stabilized with five intramolecular disulfide bonds to make an elongated globular molecule (Figure 5A). Approximately 25 residues constructing four successive loop segments construct an IBS on a region opposite to N and C termini (Figure 5A,B). The positions of the α-helices, β-strands, and disulfide bonds of AFP II are highly conserved in all canonical C-type lectins, including RSL [50]. Note that the IBS shown in Figure 5A was similarly identified for a Ca^2+^-dependent species of AFP II from Japanese smelt *Hypomesus nipponensis*, for which a high-resolution structure was recently determined for both Ca^2+^-free (6JK5.pdb) and Ca^2+^-bound states (6JK4.pdb) [51]. Their overall structures are highly identical to each other and to the present AFP II. As expected from the sequence identity, a decamer model of AFP II was readily created with software Coot [52] and Pymol [53] via molecular replacement of RSL with AFP II without any steric hindrance (Figure 5C). In this model, five AFP II molecules were situated side-by-side so as to assemble their N/C termini at a center of the disk, and two disks are stacked with each other to construct the AFP II decamer. A front view of the disk (Figure 5C, left) is made of five copies of Figure 5B pasted with 72° rotations, representing the location of IBSs at the rim of the decamer disk. The side view (Figure 5C, right) further shows that two imperfectly aligned IBSs are proximal to each other.

An IBS of an AFP species generally accompanies the hydration waters in an ice-like arrangement, for which an anchoring role to bring the host protein to a specific ice plane has been speculated [22,23,24]. An interfacial region between bulk waters and bulk ice consists of a 10 to 15 Å-thick, intrinsically disordered “quasi-liquid layer,” which is more ordered than bulk waters, but less ordered compared with the hexagonal ice lattice [54,55]. The ice-like waters of an AFP molecule were thought to merge with this quasi-liquid layer in conjunction with the ice growth. In the case of Ca^2+^-dependent AFP II from Japanese smelt, only a Ca^2+^-bound form accompanies such ice-like waters to bind to the ice first prism plane [51]. If AFP II undergoes oligomerization similar to that shown in Figure 5C, it will combine two or more IBSs to change the formation of ice-like waters with the increase in their size, which may confer an additional ability to bind to multiple ice planes. A final conclusion should be drawn after clarifying structure of the oligomerized form of AFP II, which will support, refute, or require modification of the present assumption.

To summarize, the present study determined the solubility limit of native AFPs and AFGP, and evaluated concentration dependence of their TH value and ice-binding property. These proteins are highly soluble in water, and exhibit 2.0–3.0 °C of high TH value at 50–200 mg/mL. When the ice melts to become liquid water, the hexagonal ice structure will collapse [56,57]. Suppose that some waters in 4 °C-chilled water are organized into an ice-like arrangement, they may preferably merge with the ice-like waters located on the AFP molecules.

## Supplementary Materials

The following are available online at https://www.mdpi.com/2218-273X/10/3/423/s1, Figure S1: Tricine SDS-polyacrylamide gel electrophoretogram (15%) of native AFGP sample, Figure S2: Comparison between the weight- and fluorometer-base concentrations of native AFP samples.
