# Supplementary material for: Fish-Derived Antifreeze Proteins and Antifreeze Glycoprotein Exhibit a Different Ice-Binding Property with Increasing Concentration
Source: Biomolecules. 2020 Mar 9;10(3):423. doi: 10.3390/biom10030423 (PMC7175324; doi:10.3390/biom10030423)
Supplement: Supplementary file 1 [file biomolecules-10-00423-s001.pdf]

biomolecules

MDPI

Supporting Information

Fish-derived antifreeze proteins and antifreeze glycoprotein exhibit different ice-binding property with increasing concentration

Sakae Tsuda, Akari Yamauchi, N.M.-Mofiz Uddin Khan, Tatsuya Arai, Sheikh Mahatabuddin, Ai Miura, and Hidemasa Kondo

S1 Tricine SDS-polyacrylamide gel electrophoretogram (15%) of native AFGP sample

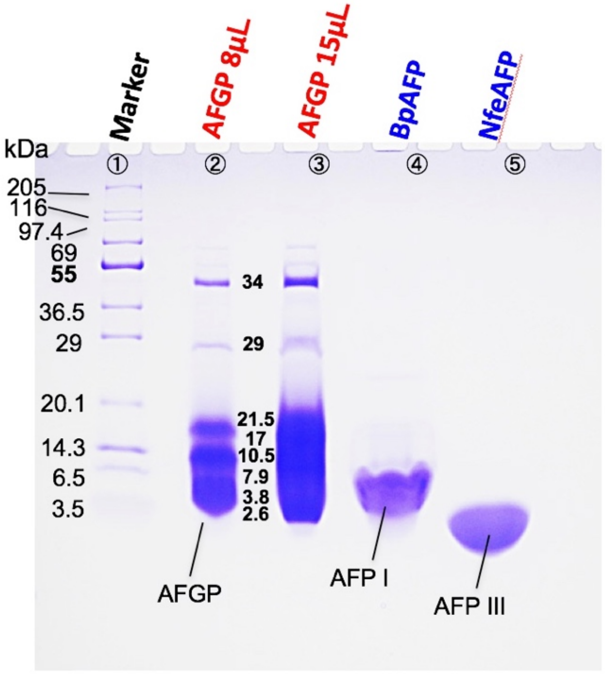

Captions to S1: Tricine SDS-PAGE (15%) of AFGP from saffron cod (*Eleginus gracilis*), AFP I from barfin plaice (*Liposetta pinnifasciata*), and AFP III from notched-fin eelpout (*Zoarces elongatus Kner*). Each protein was dissolved with 25 mM Tris buffer (pH =7.8) containing 2% SDS and 10% glycerol, and applied to a 15% precast gel (e-PAGEL E-T15L; ATTO Corporation, Tokyo, JAPAN) set into an electrophoresis tank (AE-6500; ATTO Corporation, Tokyo, JAPAN). The running buffer consists of 25 mM Tris, 25 mM tricine, and 0.05% SDS. The AFGP in eight different sizes (2.6 – 34 kDa) are detected predominantly, which are in accordance with the previous indications (ex. Raymond JA & DeVries AL, 1972, Cryobiology 9, 541-547), for which the averaged molecular weight was roughly estimated at 12 kDa.

S2

Comparison between the weight- and fluorometer-base concentrations of native AFP samples

< AFP I >

Example 1:

Weight-base concentration = 1,040 μg/mL Solvent: Milli Q (200 μL)

Sample NO.	Dilution ratio	Fluorometer reading (μg/mL)	Fluoro-base concentration	Fluoro-base Average
1	1/200	5.71	1,140	1,017 μg/mL
2	1/100	10.6	1,006	
3	1/67	15.2	1,013	
4	1/50	18.2	910	

measured after overnight incubation at 4 degC

Example 2:

Weight-base concentration = 500 μg/mL Solvent: Milli Q (200 μL)

Sample NO.	Dilution ratio	Fluorometer reading (μg/mL)	Fluoro-base concentration	Fluoro-base Average
1	1/200	2.90	580	469 μg/mL
2	1/100	4.80	480	
3	1/67	6.37	425	
4	1/50	7.83	391	

measured after overnight incubation at 4 degC

< AFP II >

Weight-base concentration = 500 μg/mL Solvent: Milli Q (200 μL)

Sample NO.	Dilution ratio	Fluorometer reading (μg/mL)	Fluoro-base concentration	Fluoro-base Average
1	1/200	3.12	624	494 μg/mL
2	1/100	5.40	540	
3	1/67	6.49	433	
4	1/50	7.57	379	

measured after overnight incubation at 4 degC

< AFP III >

Weight-base concentration = 1,000 μg/mL   Solvent: Milli Q (200 μL)

Sample NO.	Dilution ratio	Fluorometer reading (μg/mL)	Fluoro-base concentration	Fluoro-base Average
1	1/200	5.36	1072	
2	1/100	9.10	910	879 μg/mL
3	1/67	12.1	807	
4	1/50	14.5	725	

measured after overnight incubation at 4 degC

< AFGP >

Only 1/60 amount of the fluorometer-base concentration was always estimated for the solutions of native AFGP samples at the concentration of 1,000, 3,000, 5,000, and 10,000 μg/mL. The fluorescence dye may not be bound to the AFGP molecules, because of their bulky sugar moiety.

Captions to S2: Concentration measurement performed for native samples of AFP I–III and AFGP by employing a fluorometer method (Qubit Protein Assay Kit; Thermo Fisher Scientific, Waltham, MA, USA). Each AFP sample was differently diluted to prepare the samples NO.1–4 with the organic dye and buffer solution provided in this kit, and their concentration was evaluated based on their fluorometer reading. Concentration of AFGP was not determined by this method. We therefore used the weight-base concentration for AFGP, since the two concentrations exhibit consistency for AFP I–III.
